# Literary Retrospect

**Published:** 1863-07

**Authors:** 


### Art XII.—LITERARY RETROSPECT.

A very able treatise,* from the pen of Dr. Harley, adds largely to our knowledge of the pathology and treatment of jaundice.

The author begins by showing that jaundice is not a disease *per se;* but simply the most prominent symptom of several widely different pathological conditions, and in discussing it he limits himself entirely to the considerations of the opinions at present held by the more advanced of our pathologists; the object of the monograph being, not to portray the views of others, but to give a brief exposé of the author's own views, and to point out how modern physiology and chemistry have not only thrown a new light on the pathology, but have also given us a clue to the successful treatment of the disease.

He dwells on the means of distinguishing the various causes of jaundice one from another, knowing, as he says, that unless we have a clear appreciation of the cause, it is not only difficult, but even dangerous to treat the symptom. The injudicious administration of a remedy here may hasten the termination we most desire to retard. (p. 49.)

The value of analysing the urine in the various forms of liver disease is carefully pointed out, for, as the author says, "in jaundice, from suppression, the liver does not secrete bile; consequently, no bile-acids being formed, none can enter the circulation, and they are therefore not to be detected in the urine. In jaundice from obstruction, on the other hand, bile is secreted, and absorbed into the blood; and the bile-acids not being all transformed in the circulation, as Frerichs supposed, are eliminated by the kidneys, and appear in the urine, where they can be detected. The readiest mode by which the biliary acids may be detected is the following: to a couple of drachms of the suspected urine add a small fragment of loaf sugar, and afterwards pour slowly into the test-tube about a drachm of strong sulphuric acid. This should be done so as not to mix the two liquids. If biliary acids be present, there will be observed at the line of contact of the acid and urine—after standing for a few minutes—a deep purple hue. This result may be taken as a sure indication that the jaundice is due to obstructed bile-ducts." (p. 61.)

---

"Jaundice: its Pathology and Treatment. With the Application of Physiological Chemistry to the Detection and Treatment of Diseases of the Liver and Pancreas." By George Harley, M.D., Professor of Medical Jurisprudence in University College, London; Assistant-Physician to University College Hospital, &c. London: 1863. 8vo. Pp. 136.

Again, cancer in the liver may often be discovered by the presence of melanine in the urine, for, as the author says, "Four years ago (1858), Dr. Eiselt, of Prague, called attention to the fact, that in cases of melanotic cancer of the liver, melanine appears in the renal secretion. In jaundice, arising from melanotic cancer of the liver, the recognition of the presence of melanine in the urine would be an important aid to the diagnosis. Care must be taken not to confound the dark olive-green urine, occasionally met with in other forms of jaundice, with the melanotic urine just described, or both patient and doctor may become unnecessarily alarmed." (p. 68.)

Jaundice may arise in the following manner:—

FROM SUPPRESSION.

| | |
|---|---|
| Enervation . . . . . . . . | Fright, Anxiety, Over mental exertion, Concussion of brain. |
| Congestion of Liver (active) . | Hepatitis, Direct violence, Dyspepsia, Ague, Typhus, Typhoid, Scarlatina, Pyæmia, Yellow Fever, Poison. |
| Congestion of Liver (passive) . | Heart disease, Pneumonia, Pleurisy, Imperfect circulation in the new-born. |
| Absence of Secreting Substance . . . . . . . . | Cancer, Cirrhosis, Fatty and Amyloid Degenerations; Acute and Chronic Atrophy. |

FROM OBSTRUCTION.

| | |
|---|---|
| Congenital deficiency of Duct. | Small Ducts? Common Duct. |
| Accidental Obstruction in Course of Duct . . . . . . | Gall Stones, Hydatids, Foreign bodies from Intestines. |
| Closure of outlet . . . . . | Pressure of Pregnant Uterus, Impacted Fæces in Tranverse Colon, Organic Disease of Pancreas, or of neighbouring organs, Abscess in Head of Pancreas, Ulcer in Duodenum. |

Speaking of treatment, Dr. Harley observes: "After what has been said regarding the pathology of jaundice, I need scarcely remark that the treatment must vary according to the kind of disorder we have to deal with. A line of treatment, found to be beneficial in one case of jaundice might prove very hurtful in another. For, as has been shown in the foregoing pages, jaundice from suppression and jaundice from obstruction are, it might be said, two entirely different diseases, with only the symptoms of yellow skin, high coloured urine, and pipe-clay stools in common. The success of our treatment will therefore depend on our powers of diagnosis." (p. 101.)

The benefit of mercury in cases of diseases of the liver cannot, Dr. Harley thinks, be denied; but the injudicious employment of this drug in cases of jaundice has frequently been followed by the most disastrous results. There was a time when mercury was administered in all cases of jaundice, irrespective of their cause: now, however, men are fortunately becoming more careful in the employment of this drug. But there is still a mistaken notion regarding the therapeutical action of mercurial preparations. It was at one time thought that they stimulated the liver to secrete bile, and now since physiology has shown that they possess no such action, many have gone to the opposite extreme, and declared, that if mercurials do not stimulate the liver to secrete bile, their benefit in hepatic disease has been a delusion.

The action of mercurials seems to be this—"mercury is a powerful antiphlogistic; it reduces the volume of the blood by its purgative properties, and it impoverishes the blood by its direct action on the red corpuscles. It has been poetically said, by Dr. Watson, that mercury can blanch the rosy cheek to the white of the lily; and nothing is more true, for in experiments on animals, I have found the prolonged use of mercury reduce the red blood corpuscles to a minimum." (p. 103.)

After detailing the best treatment to adopt in cases of gall stones, the author says of podophyllin, "In cases of feeble liver, where there is an insufficient secretion of bile, from want of nervous power, podophyllin is decidedly of service, for in such cases mercury is, of course, counter-indicated. Moreover, podophyllin can be advantageously combined with vegetable tonics, and, when given along with gentian or quinine, forms an admirable hepatic stimulant in some of the cases usually denominated torpid liver. I cannot refrain from making a few remarks on what I consider the injudicious employment of podophyllin. In cases of jaundice, for example, podophyllin is at one and the same time the bane and the antidote. The bane in all cases of jaundice from obstruction, the antidote in a few cases of jaundice from suppression." (p. 113.)

Lastly, in cases of liver disease, where patients are gradually becoming emaciated and exhausted from the absence of the biliary secretion from the digestive canal, the author recommends supplying artificially the place of the absent bile in the digestive process. Not, however, in the way usually adopted, of giving inspissated bile along with the food; a method of treatment which originated ere modern physiology rent the veil of therapeutical empiricism; but by giving purified bile, prepared according to the author's process, in order that it may retain all its essential digestive properties, at the end of stomachal digestion; when it will, as in the healthy organism, act on the chyme at the proper moment, and thereby render it fit for absorption. In order still further to ensure the action of the bile being delayed until the food is in a condition favourable to its action, that is to say, until it is ready to pass from the stomach into the duodenum, he has had the bile, as above prepared, put into capsules, which are not readily acted on by the gastric juice. The capsules not only preserve the active properties of the bile for an almost indefinite period, but they have the advantage of most effectually preventing the patient tasting the remedy.

In a final note the author states: "It is not alone in cases of jaundice that the prepared bile may be of service, but also in the various forms of duodenal dyspepsia, so common among the literary classes, consequent upon either a deficient quantity, or an abnormal quality of bile."

We commend this treatise to our readers: it should find a place in every practitioner's library.

The Registrar-General, in his Report for 1861,* points out an easy way of remembering the progress of population in England in the last quarter of a century. Each quinquennial period, it would appear, added a million or a little more to the account. In 1836–40 the population was rising through its 16th million; in 1841–45 through its 17th; in 1846–50 through its 18th; in 1851–55 through its 19th; and in 1856–60 through its 20th. In the middle of 1861 it was estimated to have reached 20,119,496.

The vital statistics of 1861 may be thus summed up:—There were fewer marriages than in either of the two previous years; the births were more numerous than they had ever been in England; and though the year was healthy, the mortality was greater than it had been in 1860, which was still healthier.

We cull a few details from this brimming report. Of 100 men and 100 women who were married, 75 of the former and 65 of the latter wrote their names in the registers. London, both with men and women, stands highest in an educational comparison, based on the mark-test of signatures of persons marrying. The greatest amount of ignorance in the elementary art of writing is found in the following counties, where the proportion of marrying men fell below 70 per cent.:—Berks, Herts,

* *Twenty-fourth Annual Report of the Registrar-General of Births, Deaths, and Marriages in England.*

Bucks, Huntingdon, Bedford, Cambridge, Essex, Suffolk, Norfolk, Wilts, Cornwall, Hereford, Salop, Stafford, Monmouth, and North and South Wales. In the Welsh division, which includes Monmouth, the proportion ranged as low as 60 and 65. Generally the men in the northern counties have received more education than those in the southern. In Sussex, Berks, Herts, Essex, Suffolk, Norfolk, and a few other counties, the women are better educated than the men; but in the northern portion of the kingdom this anomaly is not observed. It is a very grave fact that the women are not better educated in Lancashire than they are in North Wales.

Of every 100 births *in* wedlock and *out of* wedlock, 6·3 belonged to the latter category. In London the proportion of the illegitimate was only 4·4 per cent.; in Norfolk it was 10·3; in Westmoreland (a well-educated county, where men and women can write their names), it was 10·6; in Cumberland 11·2. In Monmouth and Wales, where education is bad, the proportion of illegitimate births was 6 or 7 per cent.

The mortality was below the average in all the counties, with the exception of those that follow:—Bucks, Huntingdon, Cambridge, Suffolk, Norfolk, Salop, East and North Ridings of York, Northumberland, Cumberland, and North Wales. If equal numbers living of the sexes are taken, the deaths of males were in proportion to those of females, as 110 to 100. But in the population as actually constituted, the females have a great preponderance, and the deaths of males were 104 to 100. The death-rate was 2·163 per cent. The mean death-rate of 1852–61 was 2·221.

In the ten years, 1840–9, the mean rate of mortality in London was 2·51 per cent.; in the subsequent decenneum, 1850–9, it fell to 2·36 per cent. "It is still more satisfactory to observe that the rate of mortality *in each of the last three years*, 1859–61, has been less than the lower of the two rates that have just been mentioned. If the mortality of London were confined permanently within the limit represented by the mean rate of the last three years, the effect of that reduction would be that more than 4000 persons would survive annually, whose lives would drop under the mean rate derived from the twenty years 1840–49. And if measures that have been already adopted are not relaxed, the amount of benefit will be increased, as the population that is the subject of it is increased. But a higher standard of health than any which the present tables show is possible, and, it may be hoped, will be attained."

The four prevailing diseases of the year were typhoid fever, cholera, diarrhœa, and dysentery.

In Dr. Farr's letter on the "Causes of Death," appended to the *Report*, attention is directed particularly to deaths from burns and scalds. No less than 3053 persons (1620 males and 1433 females) died from these causes in 1861; and in the fourteen years (1848–61), 39,927 individuals were burnt alive or scalded to death in England! Of this latter number 2958 were boys and 4982 girls, from five to ten years of age. "The Druidical sacrifices," writes Dr. Farr, "the fires of Moloch, the Inquisition, the fires of Smithfield, the burnings of witches in the Middle Ages, and the immolation of widows in India, naturally excite horror in the present age. They admit of no historical palliation. Still it is evident that the lives were offered up mistakenly with a view to the removal of evil, and that the sacrifices were sanctioned by the religion or superstition of the age and people. The deaths by burning in England are ascribed to accidents, but they are none the less dreadful on that account, particularly when it is considered that the victims are often as unnecessarily exposed as moths to the flames in which they perish. A special inquiry into the circumstances of the accidents by fire and by scalding water might suggest means by which they might be effectually prevented; in the meantime, the following points deserve immediate attention:—1. Open fires, lights, and kettles of hot water, should be surrounded by good guards. This precaution is of capital importance. 2. Children, and young people, and old women, should be systematically taught the danger of fire. 3. The dresses of young children and old women should be made as much as possible of worsted or wool, which will protect them against cold as well as against fire. 4. The muslin dresses and cotton and linen clothes might very properly be starched with the chemical materials which are found by experience to render them, to a certain extent, incombustible. 5. Private houses should all be provided, on each floor of the sleeping apartments, with the means of escape in the event of the lower apartments taking fire during the night. Especial provision to be made for women and children. 6. Fireworks, powder-works, and chemical manufactories require special precaution. 7. Persons falling asleep near the fire, or near lights, (*a*) in a state of intoxication, or (*b*) in fits, are frequently burnt to death. 8. Young children drink scalding water out of the spout of the tea-kettle. This happens often in the lower classes, when the mother is out at work, and the young children are left at home alone. The means of obviating this danger are evident. 9. Special arrangements are, it is scarcely necessary to add, required for preventing the explosions of mines or of steam-engines."

The fourth detailed Annual Report of the Registrar-General for Scotland* refers to the year 1858. This is the fourth of these detailed reports which has been published since the beginning of 1861. At the same rate of production, in two or three years more, Dr. Stark will have made up the formidable arrears in the Scotch Register House. In 1858, Scotland was suffering from depression of trade, consequent upon the great bank failures of November and December, 1857. Much distress prevailed among the manufacturing and mining population, and all classes suffered more or less. As a result the marriages exhibited a great decrease, and the mortality rose higher than it had been during the three previous years. The births, not being under the same agencies, were in full proportion.

The births were in the proportion of 345 to every 10,000 of the estimated population of that year. This was considerably above the English birth-rate of the same year, which was in the proportion of 336 in every 10,000 of the estimated population. "If we compare the birth-rates of Scotland and England for the years 1856, 1857, and 1858, the same result is arrived at, viz., that the Scottish birth-rate is rather higher than the English, the mean annual birth-rate of England for that period having been 342 births in every 10,000 persons, while the Scottish was 344 in a like population. This is an extremely curious result, inasmuch as the proportion of marriages in Scotland is so much lower than it is in England." The report contains many facts illustrative of the remarkable phenomena presented by illegitimacy in Scotland.

The proportion of marriages in 1858 was in Scotland 652 in every 100,000 population ; in England, 805. The average marriage-rate of England is 820 in every 100,000 population ; of Scotland, for the four years, 1855–58, it was about 679.

The mortality in Scotland in 1858 was in the proportion of 211 in every 10,000 population ; in England it was 230. The mean annual mortality for Scotland in the four years 1855–58, was 208 in 10,000 population ; in England, in the same period, 220.

The influence of towns in augmenting mortality is brought out in the clearest light in the detailed examination of the mortality returns, and it is shown that this influence affects people of all ages. "The facts clearly establish" (says the Report) "that it is not the more unhealthy occupations nor the vices of a town, nor the abuse of intoxicating liquors or of tobacco, which cause the higher mortality in the town ; but that it is something which affects every resident at every age—

* *Fourth Annual Report of the Registrar-General of Births, Deaths, and Marriages in Scotland.*

ay, even the nursing infant more powerfully than the adult." In 1858 the proportion of persons who died in the insular districts of Scotland was 146 in every 10,000 population; in the Mainland Rural districts, 180; while in the Town districts the proportion rose to 269, or nearly double the rate of the insular districts.

The Epidemiological Society completes the first volume of its Transactions by the publication of a third part.* This part contains the report of the Council for the year 1861–2, and a report on Epidemics in 1860–61, by the late Dr. J. O. McWilliam. A paper "On an Indian Remedy for Small-pox," by Mr. H. Chalmer Miles, Assistant-Surgeon, R.A., refers to the *Sarracenia Purpurea,* or Indian pitcher plant. The Indian fame of this plant in the treatment of small-pox, was first made known by Mr. Miles to the Epidemiological Society. At first the nature of the plant was not known, and the specimens submitted to the Society were insufficient to enable an opinion to be formed of its character. Subsequently, after considerable difficulty, Mr. Miles procured other portions of the plant than those actually used medicinally, and it proved to be the *Sarracenia Purpurea.* A series of experiments, made by Mr. Marson, of the Small-pox Hospital, at the request of the Epidemiological Society, and which have recently been submitted to the Society, seem to demonstrate that the drug is inert, and its Indian reputation a vain imagination. A paper by Dr. Archibald Smith, "On the Spotted Hæmorrhagic Yellow Fever of the Peruvian Andes in 1853–57," is of great interest and importance. Mr. J. N. Radcliffe, one of the honorary secretaries of the Society, discusses the facts connected with the early history of the recent epidemic of Diphtheria; and Dr. Robert Elliot offers certain suggestions on the diseases and injuries of artisans and labourers traceable to their respective occupations. Other papers found in the part, are a "History of an Outbreak of Fever at Over Darwen in the autumn of 1861," by Dr. E. Headlam Greenhow; "Notes on the recent prevalence of Yellow Fever in several of her Majesty's ships on the West India station; and the recent introduction of the disease into the port of Halifax, with remarks on the climate, &c., of Halifax," by Dr. Slayter, Health Officer of the Port; "On the Vital Statistics of Tasmania" in 1861, by Dr. Swarbeck Hall, of Hobart Town; "Note on Diphtheria in Peru," by Dr. Archibald Smith; "On Dr. A. Hirsch's Handbook of Historico-Geographical Pathology," by Dr. Hermann Weber;

---

* *Transactions of the Epidemiological Society of London.* Vol. i., part iii. London. 8vo. pp. 175.

and a Report on Epidemics in 1861–2, in two parts; the first being devoted to an account of the geography of certain epidemics abroad, by Dr. Gavin Milroy; the second, to the state of Epidemic, Epizootic, and Epiphytic disease in Great Britain, by Mr. J. N. Radcliffe. The report refers to the twelve months from the 1st October, 1861, to the 1st October, 1862. The following is Mr. Radcliffe's summary of the state of health in Great Britain within this period:—

"Reviewing the whole of the twelve months, the following results are arrived at:—

"The health status of the English population, as estimated from the unusually low rate of mortality throughout the year, was generally good, notwithstanding the dearness of provisions and the excessive amount of pauperism. The health status of the Scottish population was remarkably inferior to its average condition, as shown by the large amount of sickness prevalent in the last quarter of 1861, and the higher rate of mortality since the commencement of the present year.

"It is well to mark, however, that the average death-rate of Scotland is below that of England. Thus, during the six years 1855-60, the annual proportion of deaths in England was 219 per 10,000 population; during the same period in Scotland the proportion was 208.

"The high range of temperature during the winter months, and low range during the summer, in England, exercised a favourable influence over the health, notwithstanding much variableness of the weather and wetness. In Scotland the same conditions of temperature and weather existed, but to an exaggerated extent; and the great changes which were experienced, and especially the undue humidity of the atmosphere, were apparently the chief fostering causes of the influenza and throat affections, which seem to have been more common than in England.

"The epidemic diseases most prevalent in England were continued fever, scarlatina, measles, diphtheria, whooping-cough, and small-pox. In Scotland the same diseases, with the exception of small-pox, and in addition and more particularly sore-throat, often assuming a diphtheric character, and accompanied by diphtheria, played the chief part in the epidemiology of the year.

"In both parts of the kingdom continued fever prevailed most commonly in the autumn quarter of 1861; and in England the affection would appear to have been more general in the northern districts. In both countries, also, scarlatina was widely prevalent in the northern counties in the last quarter of 1861; in both countries the disease has shown itself more active in the southern counties in the quarter last past.

"Measles prevailed extensively, and in some instances very fatally, in the winter quarter, in England, and, so far as may be judged from the data, chiefly in the two opposite extremities of the kingdom; in Scotland the disease appears to have been most prevalent in the spring and summer quarters of the present year.

"Diphtheria in England was principally fatal in the autumn quarter, but it has prevailed more or less in every registration district during the twelve months. In Scotland the disease, together with sore-throat, appears to have been epidemic throughout the year.

"Whooping-cough was widely prevalent in England during the autumn and winter quarters; in Scotland during the winter and spring.

"Influenza was epidemic in Scotland in the first half of the year.

"Finally, small-pox broke out in many districts of England, but more especially in the Eastern, and South-Western and Northern Counties, and in Yorkshire.

"None of the outbreaks of the diseases referred to as occurring in England, at any time during the twelve months assumed what may be termed general proportions: they were emphatically local irruptions. But the dispersion of the various maladies, or of their centres of manifestation over the kingdom; the cropping-out or exaggeration in different localities; and the effects apparently exercised upon the sickness and mortality of different districts without heightening that of the whole kingdom above the average, present a study of no ordinary interest. From this study we are probably justified in concluding that it is in dealing with these local outbreaks of disease, and the conditions on which they depend in ordinary periods, that our best chance consists of warding off the wider spread and more deadly outbreaks of extraordinary periods.

"The detailed mortuary returns of Scotland extend as yet only to the year 1857; but the returns for England are brought down to 1860. From the latter, then, we may obtain information as to the status of the epidemic diseases referred to, in this country, almost immediately preceding the period I have discussed.

"Since 1857 the mortality from continued fever has slowly declined. In that year the deaths from this cause were 19·016; in 1860 they amounted to 13·012.

"In 1855 the mortality from scarlatina was 17·314. In 1856-57 the number of deaths from this disease fell considerably; in the latter year the mortality being 12·646. The year following the number of deaths increased enormously, becoming well-nigh doubled—the amount being no less than 23·711. In 1859 the number fell to 19·310; and in 1860 it became as low as 9·305. Prior to 1855 deaths from scarlatina, cynanche maligna, and diphtheria, are not separated in the Registrar-General's reports. Whether the detailed reports of the Registrar-General will show an increase of the mortality in England from scarlatina during 1861 and 1862 as great as has occurred in London, cannot be predicted. It is certain, however, that the activity of scarlet-fever has been considerable in many parts of the kingdom.

"The deaths from cynanche maligna in 1855 amounted to 199; in 1858 to 1770. In 1860 the mortality from the disease had decreased to 376.

"The mortality from measles was largely augmented in 1858, and there was a steady increase in the number of deaths from the disease in the two subsequent years.

"The deaths registered from diphtheria in 1855 numbered 186; in 1859, 9587. In 1860, the mortality from this disease had decreased to 5,212.

"The mortality from whooping-cough in 1860 was the lowest since 1852.

"The mortality from small-pox had declined from 6460 in 1858 to 2749 in 1860.

"The reduced rate of mortality throughout England which occurred in 1860, was chiefly due, Dr. Farr remarks, to the decline of the number of deaths from scarlatina, diphtheria, and diarrhœa. The decrease in the deaths from small-pox, erysipelas, and cholera, also contributed to the favourable result."

A second edition of Mr. Charles Bray's work on the "Philosophy of Necessity"* lies before us. The object of the author, as stated in the introduction, is to pursue the inductive method of inquiry in investigating the nature of man, his place in creation, the character of his mind, and particularly to trace to its legitimate consequences the doctrine of philosophical necessity, which the connexion between cause and effect implies. He would show that the mind of man is not an exception to nature's other works; that, like everything else, it has received a determinate character; that all our knowledge of it is precisely of the same kind as that of material things, and consists in the observation of *its order* of action, or of the relation of cause and effect. This is a truth which, although acknowledged by many writers, has never yet been made of sufficient importance in the science of Mental and Moral Philosophy. It has either been considered as a mere abstract of no practical use, or else avoided and stifled as leading to fatalism, and otherwise dangerous in its tendency. But he hopes to be able to show, on the contrary, that upon this truth *alone*, however it may be said to militate against man's free-will or accountability, in some acceptation of the terms, our educational and political systems can be properly based in accordance with the nature of the being to be educated and governed. If in setting a steam-engine to work, the engineer were to leave much to *free-will*, the work would be but badly performed: So, as relates to man, if in our educational systems, the causes are inadequate to the intellectual and moral results we derive, his *free-will* will not supply the deficiency. (p. 6.)

Mr. Curling publishes a third edition of his valuable work on Diseases of the Rectum.† Eight new chapters are added to the

---

* *The Philosophy of Necessity; or Natural Law as applicable to Moral, Mental, and Social Science.* By Charles Bray. Second Edition, Revised. London. 8vo, pp. 446.

† *Observations on the Diseases of the Rectum.* By T. B. Curling, F.R.S., Surgeon to the London Hospital. Third Edition, Enlarged and Revised. London, 8vo. pp. 232.

work, and a great part re-written. The subjects comprised in the new chapters are nervous affections of the rectum, the villous tumour, epithelial cancer of the anus and rectum, atony of the rectum, organic contraction of the anus, and operations for their relief, congenital imperfections of the anus and rectum, and colotomy in cases of imperforation.

A second edition of Dr. Hassall's work on the Urine* will be warmly welcomed. The work is in every respect a new one, and from its handy size, yet comprehensive character, is admirably adapted for use. Not the least of its merits is a profusion of illustrations, coloured, when requisite, by the hand.

* *The Urine in Health and Disease: being an Exposition of the Composition of the Urine, and of the Pathology and Treatment of Urinary and Renal Disorders.* By Arthur Hill Hassall, M.D. London. Second Edition, small 8vo. pp. 416.

